# Decline in Size-at-Maturity of European Hake in Relation to Environmental Regimes: A Case in the Eastern Ionian Sea

**DOI:** 10.3390/ani14010061

**Published:** 2023-12-23

**Authors:** Aglaia Legaki, Archontia Chatzispyrou, Dimitrios Damalas, Vasiliki Sgardeli, Evgenia Lefkaditou, Aikaterini Anastasopoulou, Aikaterini Dogrammatzi, Konstantinos Charalampous, Caterina Stamouli, Vassiliki Vassilopoulou, George Tserpes, Chryssi Mytilineou

**Affiliations:** 1 Hellenic Centre for Marine Research, Institute of Marine Biological Resources and Inland Waters, 16452 Athens, Greecekostash@hcmr.gr (K.C.); chryssi@hcmr.gr (C.M.); 2 Hellenic Centre for Marine Research, Institute of Marine Biological Resources and Inland Waters, P.O. Box 2214, 71003 Heraklion, Greece; shark@hcmr.gr (D.D.); vsgard@hcmr.gr (V.S.); gtserpes@hcmr.gr (G.T.)

**Keywords:** *Merluccius merluccius*, maturation, climate change, regime shift, Mediterranean Sea

## Abstract

**Simple Summary:**

The European hake is under fishing pressure, both in the Atlantic and the Mediterranean waters. Moreover, in certain geographic locations, its size-at-maturity has been reported to decline over time. Understanding biological processes in marine life can assist in species stock evaluation and future management, allowing for the avoidance of population decline and diversity loss. This work focuses on exploring the variation in European hake size-at-maturity in the eastern Ionian Sea (Central Mediterranean) during the last five decades, investigating, at the same time, its relationship with environmental change.

**Abstract:**

European hake, *Merluccius merluccius* L. 1758, is a highly valuable demersal fish species exploited in both the east Atlantic and the Mediterranean Sea. Changes in the size-at-maturity of this species have been reported in various geographic areas. Size-at-maturity is a key parameter in fishery management. Our main goal was to study the trend of the size-at-maturity of European hake in the eastern Ionian Sea (Central Mediterranean) over the last five decades. Utilizing a multi-decadal series of data for various environmental variables, we employed multivariate analyses and non-additive modeling in an attempt to identify shifts in the climatic environment of the eastern Ionian Sea and whether the maturation of the hake population could be affected by these changes. The analyses used suggest a plausible environmental regime shift in the study area in the late 1990s/early 2000s. The decrease in size-at-maturity that was detected in the last two decades may, thus, be associated with environmental changes. However, as many fish stocks already experience fishery-induced evolution, further investigation is necessary to determine whether this environmental effect is an additional stressor on a possibly already fishery-impacted population. The outcomes of this study highlight the importance of investigating the relationship between fish reproductive traits and altered environmental conditions, as the latter are generally ignored during assessments, affecting the robustness of fishery management.

## 1. Introduction

Life-history traits represent one of the primary determinants of population dynamics, and their evolution affects stock biomass, demography and economic yield [1]. Among them, size-at-maturity (the estimation of the size at which 50% of individuals are mature—*L*_50_) is applied to set the minimum conservation reference size (MCRS) for exploited stocks, which is important in fishery management. Yet, *L*_50_ may fluctuate temporally, and using it as a constant parameter over long periods of time may invalidate estimates of stock status; the need to periodically update *L*_50_ is, therefore, essential. Moreover, it has been documented that size-at-maturity is linked to population size. According to the compensatory growth theory [2], at low population levels, fish are more likely to mature at an earlier age as the reduction in population density increases food supply. Such population changes can be caused by natural (environmental drivers—climate change) or anthropogenic pressures (the removal of individuals by fisheries). Furthermore, the growth and reproduction of fishes are linked with favorable environmental conditions [3]. For fish that live in habitats with noticeable ambient condition changes, their reproductive strategy adjusts to environmental fluctuations to ensure offspring survival [4]. Previous studies have reported declines in the age- or length-at-maturity in many fish stocks [5,6,7]. Size at first sexual maturity is under strong evolutionary selection pressure in fish, and possible evolutionary changes (because of the selection of certain genotypes) toward a smaller maturation size have been detected due to intense fishing pressure and/or environmental changes [8,9,10].

Environmental volatility and climate change in general can affect fish in many ways, such as triggering physiological responses and altering productivity [11]. When the effects of these drivers are prolonged, they could even lead to evolutionary changes, shaping a new population status [12]. Such changes in the environment may occur over long periods of time and can be documented only with a multi-annual time series of data. In certain cases, these changes can steer the whole ecosystem towards a new and stable state, and recovery to its previous state may not be feasible. Such reorganizations of ecosystems are known as “regime shifts” [13,14]. Regime shifts are described as abrupt changes in the ecosystem state and can even be triggered by smooth, continuous climatic shifts [14], particularly in ecosystems with low ecological resilience, as a result of anthropogenic pressures, such as fishing, pollution and habitat degradation [15]. In the eastern Mediterranean Sea, regime shifts in oceanographic variables and fishery resources have been documented over the past few decades [16,17], related to ecosystem reorganizations and discontinuous responses to sea warming.

European hake, *Merluccius merluccius* L. 1758, is a highly valuable demersal fish species exploited in both the east Atlantic and Mediterranean Sea. It is widespread, from Norway and Iceland and southward to Mauritania, including the Mediterranean and Black Seas [18]. Research on the population dynamics of European hake in the Mediterranean revealed the species’ preference for a water temperature between 14 and 18 °C [19]. The species is characterized by a prolonged spawning period, extending throughout the year [20], with multiple spawning peaks varying between areas ([21] and references therein). Size-at-maturity has been investigated thoroughly in the Mediterranean [21,22,23,24,25], with values for females ranging from a minimum of 215 mm in the eastern basin—Izmir Bay [24]—to a maximum of 465 mm in the northern Tyrrhenian Sea [26].

In the eastern Ionian Sea, the official annual landings for this species have fluctuated between 500 and 1800 tons over the past 20 years [27]. Various fishing techniques are involved in hake fishery; the bulk of catches come from static nets (73%), followed by bottom trawlers (19%) and static longlines (8%) [27]. European hake is generally considered overfished in most regions of the Mediterranean Sea, with an average fishing mortality (*F*) more than three times higher than fishing mortality when stock is kept within safe biological limits (*F_MSY_*) [28]. The most recent attempt to assess hake population status in the eastern Ionian concluded that the species is in overexploitation, with intermediate biomass [29]. However, these results are uncertain due to several chronological gaps in the data used to estimate the population dynamics of European hake within the past 20 years of research. Hence, being aware of this issue, fishing pressure is considered one of the key factors related to biological changes observed for the species in the eastern Ionian Sea. Nevertheless, as environmental data were recorded for a time series of 60 years, using this robust dataset for investigating the relationship between environmental parameters and the ‘biological system’ (i.e., the size-at-maturity of hake) seemed quite relevant for shedding further light on the complex interactions existing in marine ecosystems.

Therefore, the objectives of the present work were (i) to examine the trend of size-at-maturity of European hake in the eastern Ionian Sea over the last few decades, (ii) to identify shifts in the climatic environment of the study area and (iii) to investigate whether the observed European hake population metric (size-at-maturity) could be associated with climatic shifts. We applied this approach to a multi-decadal time series of environmental variables and size-at-maturity estimates. Our aspiration was to understand whether large-scale environmental changes may play a role in hake reproductive strategy at a regional scale.

## 2. Materials and Methods

### 2.1. Study Area

**Figure 1:**
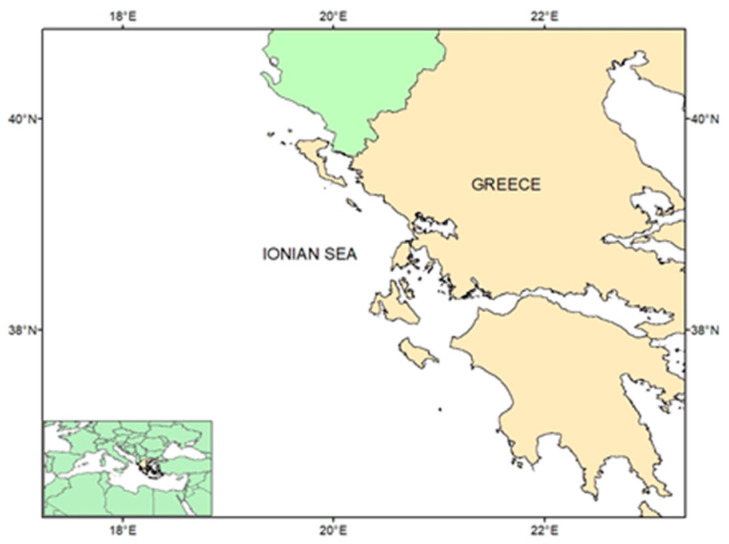
Map of the study area in the eastern Ionian Sea (GSA20).

The study area extends into the main part of the eastern Ionian Sea (central Mediterranean) at depths ranging between 20 and 800 m (Figure 1). The eastern Ionian is the deepest basin of the Mediterranean Sea, reaching a maximum depth of 5121 m. The oxygen and nutrient patterns are affected by the presence of mesoscale cyclonic and anticyclonic gyres in the area, which is characterized as a highly oligotrophic environment where phytoplankton growth depends mainly on regenerated nutrients [30], as well as by larger-scale changes regarding intermediate- (60–900 m depth) and deep (>700 m depth)-water-mass thermohaline circulation, known as the Eastern Mediterranean Transient (EMT) of climate forcing [31]. The zooplankton abundance is higher in the northeastern Ionian Sea compared to that in the central and southern areas, which are considered among the most oligotrophic regions of the Hellenic waters [30]. The study area is included in the geographic subarea GSA 20 (Figure 1) of the General Fisheries Commission for the Mediterranean (GFCM).

### 2.2. Data

#### 2.2.1. Biological Data (Size-at-Maturity)

The total length (TL) and sexual maturity data of the European hake (hereafter ‘hake’) in the eastern Ionian Sea (GFCM GSA 20) were collected from six research projects of the Institute of Marine Biological Resources and Inland Waters (Hellenic Centre for Marine Research—HCMR, Greece) and in the framework of the National Fisheries Data Collection Programme (NDCP/DCF) (see data sources in Table S1 of the Supplementary Material). Data from different types of fishing gear were combined in order to increase the number of individuals and the range of individual sizes available for the analysis, as static gear, i.e., nets and mainly longlines, usually exploit large specimens. However, the main bulk of the samples (85%) were derived from bottom trawl. These data were retrieved from the IMBRIW database [32], for the period between 1983 and 2021 (with some gaps between years). Gaps in the time series of data were related to the absence/limited data of research projects and due to limited implementation of the National Programme (NDCP/DCF). From the total set of data, only female data were elaborated upon, since the use of female *L*_50_ is preferred for fishery management purposes (e.g., for defining the minimum conservation reference size). The number of female individuals per year used in our analysis (Table S2, Supplementary Material) created a sizeable dataset of 7141 individuals containing important information over the past 40 years.

Maturity was determined based on macroscopic examination of the gonads in every project included in the analysis. The Nikolsky scale (1963) [33] (six-stage key) was used in most cases, except for the MEDITS program between 1995 and 2008, when a four-stage maturity key was applied (MEDITS scale [34]). Since the estimation of *L*_50_ is based on the number of mature and immature individuals, the maturity stages of the two above-mentioned scales were converted to the categories mature and immature as follows: stages 3 and 4 of the MEDITS scale and stages 4–6 of the Nikolsky scale were considered to be mature, whereas the rest stages were considered to be immature.

#### 2.2.2. Environmental Data

Historical physicochemical and biological variables (Table S3 of the Supplementary Material) for the eastern Ionian Sea were compiled for the period 1960–2021. Data were made available through the Horizon 2020 CERES project (http://ceresproject.eu/ accessed on 10 May 2023) [35]. They were derived from the POLCOMS-ERSEM coupled model framework, producing daily, monthly and annual outputs of physical, chemical and biological variables at a 1/10 of a degree spatial resolution. POLCOMS (Proudman Oceanographic Laboratory Coastal Ocean Modeling System; [36]) is a 3D community physical model with the ability to run in regions that include both the deep ocean and the continental shelf. ERSEM (European Regional Sea Ecosystem Model; [37]) is one of the most complex lower-trophic-level marine ecosystem models currently in use, including bacteria, four phytoplankton and three zooplankton functional groups, a fully resolved diurnal cycle, variable carbon-to-chlorophyll ratios and independent nutrient pools for carbon, nitrogen, phosphorous and silicate. The POLCOMS-ERSEM modeling system is well established in the NE Atlantic and has been applied successfully in the Mediterranean [36,38,39,40]. Model outputs have been validated through comparisons to satellite values. A full description of data specifications can be found in CERES (2018) [41].

To shortlist the environmental variables of interest for this study, we focused on their relevance to size-at-maturity. From the full set of 32 environmental variables, some refer to the sea bottom (e.g., *ben_DOC*—benthic dissolved organic carbon); others are, by definition, two-dimensional (e.g., optical depth); while most of them were available in zones of different depths (3D variables, e.g., gross primary production). It has been documented that surface oceanic features can have a significant effect even on demersal resources, such as hake [19,42,43]. As a result, sea surface temperature is commonly used as a proxy for preferred temperature for demersal species (e.g., Ref. [44] and references therein on the Mean Temperature of the Catch—MTC). Moreover, it is considered to depict the warming effect on marine ecosystems, including demersal species, such as hake (e.g., [45] and references therein). Consequently, it was decided to use yearly averages on the surface over the whole eastern Ionian Sea area. To avoid duplicating information, correlation testing [46] was used to remove highly correlated variables (Figure S1 of the Supplementary Material), ending up with a subset of 12 variables: *T*, *S*, *ssh*, *grossPP*, *Z4c*, *Z5c*, *chl4*, *pH*, *O*, *ben_sus* and *ben_DOC*, *MLD* (full description in Table 1).

### 2.3. Statistical Analysis

#### 2.3.1. Biological Data Analysis

The estimation of *L*_50_ for hake each year was based on the data of mature and immature female specimens per length class from samples collected using various types of fishing gear, all year round. The use of different types of fishing gear is not expected to affect our analysis since size-at-maturity is a population characteristic, affected mainly by the ratio of mature/total individuals per length class. Moreover, in this study, the comparison of the length frequency of hake from bottom trawl (85% of samples) and that from all gear combined each year did not reveal statistically significant differences (Kolmogorov–Smirnov test, *p*-value > 0.05) (Table S4 of the Supplementary Material). Although it is suggested that *L*_50_ estimation should be based on data collected during the main spawning period of a species in order to avoid bias [47], this might be difficult for species with prolonged spawning periods. Hence, due to the protracted spawning period of hake in the study area [48] and elsewhere [21], the maturity ogive estimation in this work was fitted based on data collected all year round. Similar methodologies have been applied for the *L*_50_ of European hake in previous research [10,49]. The size of female data analyzed for *L*_50_ ranged between 100 and 870 mm in total length; however, throughout the years, the majority of sizes were between 200 and 500 mm TL (65–89%). Data were classified into size classes of 20 mm. The maturity ogive study was based on the use of a logistic model. The statistical package STATGRAPHICS was applied to fit the logistic model to the data, estimating the parameter *L*_50_ and its confidence intervals.

#### 2.3.2. Environmental Data Analysis

In order to assess the changes in the 12 environmental variables in the eastern Ionian Sea over the period 1960–2021, the difference from the overall mean of each variable was examined visually for each year. However, instead of studying the effect of each oceanographic parameter separately (or their interactions), as the whole “oceanographic” system (biotic and abiotic) is complex and multivariate, we decided to group the variables to examine the entire system/regime (hereafter called an environmental ‘stressor’), and how its change through time may have affected the *L*_50_ of hake. For this purpose, we reduced the complexity of the multivariate dataset into a single indicator (‘stressor’) by means of a multivariate analysis. Principal component analysis (PCAs) was applied based on the 12 environmental variables. PCA converts correlated variables into linearly uncorrelated variables (principal components—PCs) through the orthogonal transformation of data to a new coordinate system. To identify the key variables driving the stressor trend through time, the contributions of all original variables to the PC scores (loadings) along the first and second principal component axes (PC1 and PC2) were calculated [50,51]. The temporal development of each variable was depicted through a ‘traffic light’ plot [52,53,54], during which variables were sorted according to their loadings on the PC1 axis, raw values of each variable were categorized into quintiles and each quintile was given a specific color. The color scale included five levels of colors. The lowest and highest quintile values per variable were indicated in green and red. Although size-at-maturity data covered the period 1983–2021, the environmental ‘stressor’ index for the same period was derived from a larger dataset (1960–2021); running a PCA on environmental data from 1983 onwards would result in having a starting point (1983) lacking all previous years’ information, largely affecting the overall trend of the index. This was confirmed by running two separate PCAs: 1960–2021 and 1983–2021 (Figure S2 in the Supplementary Material). Using a lengthy time series of environmental data would allow us to identify the contrast in the change that occurred (if any) throughout the timespan of this study.

Consequently, the size-at-maturity multi-annual index (*L*_50_) was regressed against the environmental single ‘stressor’ indicator using additive (continuous) statistical models (GAMs). Generalized additive models (GAMs—[55]) are applied whenever the functional relationships between population metrics and explanatory variables are assumed to be non-linear. Herein, the expected values of the response variable (*L*_50_) were related to the predictor variables *Z_m_* (PCA-derived environmental stressor) according to the following general formulation:

$$fL_{50})=c+\sum_{m}^{}S_{m}(Z_{m_{}})$$

where *f* is the link function, *c* is the intercept, *S_m_*() is the one-dimensional smooth function of covariate *Z_m_* and *m* denotes the number of predictor variables.

All analyses were carried out in R version 4.1.3 [56] using the packages *energy* ([57]—correlation analyses), *vegan* ([58]—PCA) and *mgcv* ([59]—GAMs).

## 3. Results

### 3.1. Size-at-Maturity

**Figure 2:**
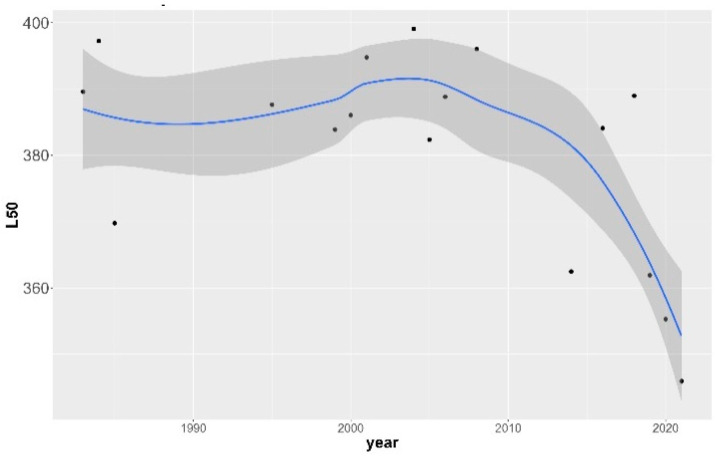
Non-linear relationship of European hake annual size-at-maturity (*L*_50_) with year in the eastern Ionian Sea for the period 1983–2021. Gray shaded area indicates 95% confidence intervals around the estimates. Black dots indicate original values.

**Figure 3:**
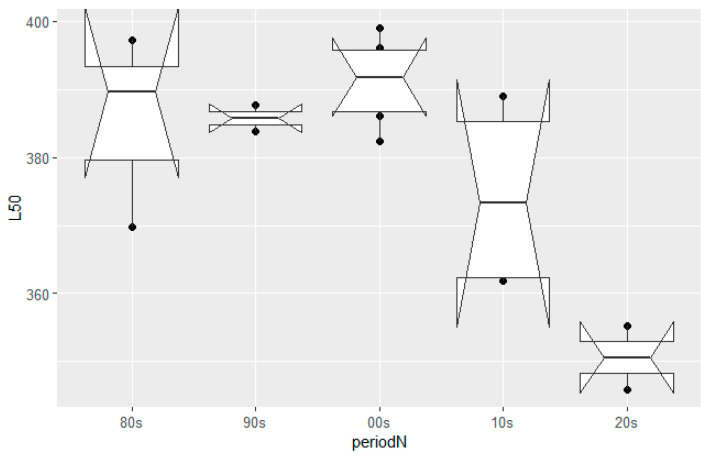
European hake size-at-maturity (*L*_50_) trend by decade in the eastern Ionian Sea from the 1980s to the 2020s. Mid horizontal line in boxplots: median; upper–lower horizontal lines in boxplots: interquartile range; dark dots: outlying points; notches: 95% CIs around the median.

The size-at-maturity (*L*_50_) historical trend for female hake in the eastern Ionian Sea was assessed by year (Figure 2) and decade (Figure 3). The logistic curves and the values of *L*_50_ across the studied period and their confidence intervals are shown in Figure S3 and Table S5 of the Supplementary Material, respectively. Two periods can be distinguished: an unvarying steady state from the 1980s up to the 2000s where size-at-maturity occurred, on average, at around 390 mm TL, followed by a period (>mid-2000) where size-at-maturity declined sharply, reaching lengths of 350 mm; this was a highly statistically significant difference (ANOVA test, *F*-ratio 13.66, *p* = 0.0022 < 0.01). The point in time where the first major changes in the population occurred was detected during the mid-2000s.

### 3.2. Environmental ‘Stressors’

**Figure 4:**
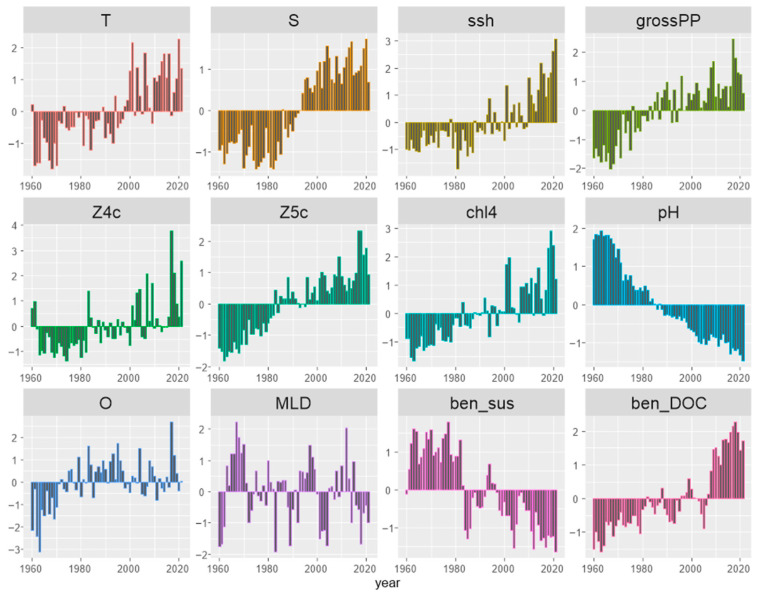
Anomalies of environmental variables in the eastern Ionian Sea during 1960–2021, expressed as difference from the overall mean divided by the standard deviation.

Visual inspection of the anomalies (Figure 4) and traffic light plots (Figure S4 in the Supplementary Material), illustrating the temporal changes in all stressor variables, suggested that most variables exhibited coherent trends in the period 1960–2021. Most prominently, *pH* exhibited a continuous decrease, while productivity-related variables (*grossPP*, *chl4*, *Z5c*, *ben_DOC*) exhibited a continuous increase.

**Figure 5:**
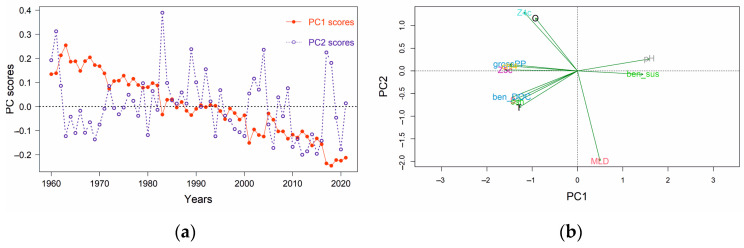
Principal component analysis (PCA) of the environmental stressors for the eastern Ionian Sea covering the period 1960–2021: (**a**) scaled annual PC scores for the first two principal components versus time; (**b**) unscaled loading plot of environmental variables in PCA.

Based on the PCA outputs, the first two PCs (PC1 and PC2) explained a relatively high proportion of the total variability: 64% and 11%, respectively. From the PC loadings, *Z5c*, *pH*, *grossPP* and *chl4*, were the most important variables across the PC1 axis (Table 2). The multivariate temporal development of the stressors during this period was visualized by plotting the annual trends of PC1 and PC2 (PC scores versus time—Figure 5a). A gradual transition along the *x*-axis during the 1990s is apparent, also demonstrated by the PC scores of each year along the PC1 and PC2 axes on a two-dimensional system (Figure S5 in the Supplementary Material). This transition was due to the contrasting loadings of different stressors on PC1 (Figure 5b) showing that among them, *Z5c*, *grossPP* and *chl4* negatively affected the PC1 value, while the effect of *pH* and *ben_sus* was positive.

### 3.3. Maturity–Environmental Stressor Relationship

Assuming a Gamma distribution with a log link function for the underlying dataset, the relationship between *L*_50_ and PC1 during 1983–2021 took the following form:

*f*(*L*_50_) ~ *c* + *s*(PC1, *k* = 7)

where *c* is the intercept, *s*() is the one-dimensional smooth function of covariate PC1 and *k* sets the upper limit of the degrees of freedom for the smoother. As PC1 was the major ‘environmental stressor’ (64%) compared to PC2 (11%), only PC1 was used for the model.

**Figure 6:**
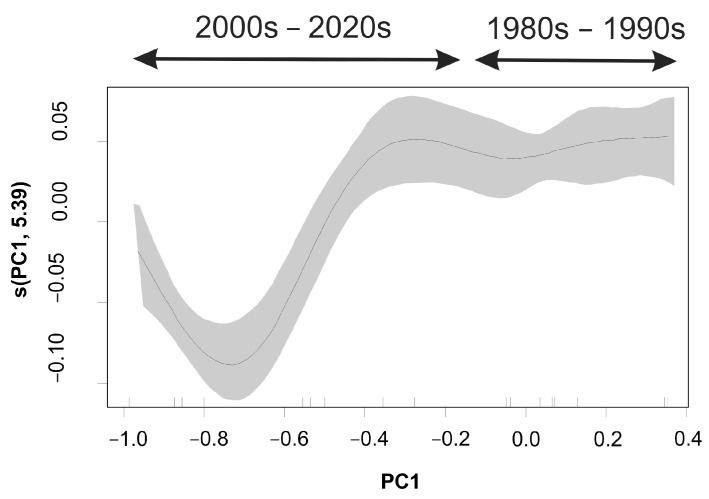
Generalized additive model (GAM)-derived effects of the environmental ‘stressor’ (PC1 scores) on the size-at-maturity *(L*_50_) of European hake in the eastern Ionian Sea during 1983–2021. Gray shaded area indicates the standard error above and below the estimates shown as solid lines.

The model explained a considerable percentage (80.2%) of the variance in size-at-maturity evolution over time (Table 3). The likelihood of a hake maturing at a larger size was higher during the early years (<2000); in the most recent period, size-at-maturity was significantly lower (Figure 6).

## 4. Discussion

Size-at-maturity constitutes an important tool for assessing and managing fish stocks [60], due to the effect of variations in productivity, both in terms of the quantity and quality of the batch [49]. In this work, the changes in the size-at-maturity of European hake and various environmental variables in the eastern Ionian Sea were examined during the last five decades to investigate potential effects linked to climate change. Our analysis revealed a decline in *L*_50_ of approximately 40 mm through a time series of 40 years. The existing gaps in the time series of maturity data did not importantly affect the analysis, and clearly, the declining trend was statistically significantly higher after the 2000s than in the previous two decades. However, this trend should also be verified in the years to come. Considering differences between sexes in response to fishery selection pressure, it has been suggested that males are more susceptible to external factors than females [61,62]. Thus, it would be interesting to investigate hake male maturation to provide a more complete picture of how the hake population reacts to changes.

The trends of maturation at a smaller size have been well documented for hake in different parts of European waters. Female *L*_50_ values of this species from the Atlanto-Iberian waters decreased by 109 mm from 1982 to 2019 [10], while in the Bay of Biscay, a decrease of 150 mm was observed from 1987 to 2004 [49]. In the case of female European hake on the Galician coast, however, a more complicated pattern was reported: a decrease of 160 mm occurred from 1980 to 1988, followed by an increase during the period 1989 to 1998, leading to a more steady period up to 2004 [49]. Furthermore, a decline in female hakes’ *L*_50_ values has been suggested by Habouz et al. [63] in the central Moroccan Atlantic as well, throughout a period of ten years, from 465 mm in 1992 [64] to 338 mm in 2002–2003 [63]. Carbonara et al. [21] have also suggested that in the Mediterranean, the recent hake *L*_50_ values are lower than those in the past (1990s). Maturation at smaller sizes and ages, compared to the past, has been recorded in various species (e.g., Atlantic cod [8]; haddock and whiting [65]; pikeperch [66]; European smelt [67]; and other species [68]).

Our multivariate analyses suggest a plausible environmental regime shift in the eastern Ionian Sea in the late 1990s/early 2000s, revealing significant changes in many biotic and abiotic parameters. The majority of the environmental parameters (biotic and abiotic ones) exhibited strikingly increasing trends within this period, during which values started exceeding the global historical mean. Zervakis et al. [69] suggested that the drastic changes in the eastern Mediterranean climatic regime in the early 1990s are attributed to processes initiating in 1986/1987, which were intensified after 1992/1993. Damalas et al. [17] also detected environmental regime shifts in the Aegean Sea in the late 1980s, followed by additional shifts in the early 2000s.

Among the factors that seem to influence marine ecological processes, temperature has been suggested to be the most influential climatic variable [70], but also seems to be the most studied [10,49,71,72]. Furthermore, the warming of seawater has been linked to decreased size-at-maturity in fish [68,73]. In addition, salinity is an abiotic factor that influences fish growth (e.g., in *Merluccius productus*; [74]) through changes in metabolic rate, food intake/conversion and hormonal stimulation [75]. Size-at-maturity appears to decrease with increasing salinity in some euryhaline fish living in high-salinity habitats [76]. It is also well known that there is wide variability in the degree of impairments among species due to the reduction in seawater *pH*, known as ocean acidification, depending on the tolerance of their physiological traits [77]. Recently, Nagelkerken et al. [78] have found that ocean acidification affects fish reproduction via indirect ways, such as increased energy budgets and enhanced reproductive-related behavior through increased primary production and food; however, the direct effect of *pH* on gonadal development was not statistically significant. Food availability, which is affected by productivity-related variables such as *Z5c*, *ben_DOC*, *grossPP* and *chl4*, has also been proven to importantly affect fish maturation; increased food availability may lead to higher growth and better body condition, and as a result, allow fish to mature at a younger age [67,79]. Trippel [6] explained simply that in scenarios of lower competition, when food intake is greater for each fish, growth will be faster and maturation will happen at a younger age. In the present study, the changes in *T*, *S*, *pH*, *Z5c*, *ben_DOC*, *grossPP* and *chl4* in the late 1990s/early 2000s coincided with *L*_50_ changes in hake. This may be indicative of a potential relationship between these factors with the earlier maturation of hake. However, Flynn et al. [80] mention that single-stressor studies may not be able to predict multiple stressor impacts on fish physiology. Hence, our approach to studying the effects of various biotic and abiotic variables on hake maturity as a combined variable (PC1—environmental ‘stressor’) was considered to be the most appropriate.

The results of non-additive modeling showed that 80% of the deviance in the detected change in the size-at-maturity of hake in the eastern Ionian Sea is explained by a combination of several environmental factors. Therefore, there is evidence that hake maturation is most likely related to long-term environmental change, indicating a potential climate-induced decrease in size-at-maturity, which has been more conspicuous during the last two decades. Ιn the NW Mediterranean, Ferrer-Maza et al. [81] suggested that European hake is characterized by plasticity, and can adjust to environmental conditions and even change its spawning strategy to meet the requirements of its habitat (alterations in biotic or abiotic parameters such as food availability, salinity or sea temperature). In the Atlanto-Iberian Waters, during the period 1982–2019, the earlier maturation of female *M. merluccius* has been related to environmental stressors, such as the North Atlantic Oscillation (NAO), as well as the level of stocks spawning biomass [10]. Shifts in the environmental regime, NAO and upwelling are also suggested to have contributed to maturation changes in hake from the Galician coast, but with an increasing trend of *L*_50_ during the period 1989–1998 [49].

The maturation of hake may be influenced by a combination of demographic, anthropogenic and environmental factors. Evolutionary theory advocates that commercial fisheries which select based on size could reasonably favor the genotype for maturation at smaller sizes, given that rapidly maturing individuals will be more likely to reproduce prior to capture [82]. A deeper understanding of the factors explaining the decrease in *L*_50_ of the studied species also requires time-series of data that are not available to date (e.g., fishing mortality and biomass estimates). Dominguez-Petit et al. [49] found that the decline in female hake size-at-maturity in the Bay of Biscay during 1987–2004 was well predicted by fishing mortality, age diversity and environmental factors (NAO and upwelling), with the latter playing the most important role in the analysis based on multiple regressions, including both environmental and fishing factors. Moreover, the same researchers suggested that the decline in female hake size-at-maturity on the Galician coast during 1980–1988 was explained by a biomass decrease related to fishing mortality. This was followed by an increase in *L*_50_ related more to environmental stressors, as mentioned previously. Therefore, ideally, changes in size-at-maturity should be linked to both environmental drivers and fishing pressure.

Unfortunately, data to assess fishing mortality or even plain fishing efforts in Greek waters are available from 2003 onwards. This would elucidate what shaped the hake population only during the last 15 years or so. Moreover, the Greek fleet has undergone some dramatic changes during the study period. As a result of over-funding in the early 1980s and subsidies for mass decommissioning after the 1990s, the fleet size and capacity increased sharply during the 1980s and contracted by more than 45% afterwards (>mid-1990s), from more than 22,000 vessels in 1995 to almost 12,000 vessels in 2022 (Greek Fishing Fleet Annual Report 2022 [83]) (Supplementary Material Figure S6). Official evaluations conducted by the scientific groups responsible for assessing stocks in the Ionian Sea (EU STECF, GFCM), based on data collected after 2003, concluded that currently, hake can be considered overfished [29], an estimate associated with a high level of uncertainty [27]. As a result, historical fishing pressure on hake in the eastern Ionian Sea prior to 2003 is largely unknown. Although fishing has most likely contributed to the observed *L*_50_ shifts, the coupling observed in the dynamics of biological and environmental stressor systems provides strong evidence that environmental change has been an important driver as well.

The main change identified herein (the early 2000s) occurred during a period when fishing capacity (and presumably fishing intensity) decreased (1998—present) (Supplementary Material Figure S6). We would expect that a reduction in fishing pressure during the last two decades would benefit the population and lead to a population increase and subsequently to an increase in size-at-maturity (compensatory growth theory). On the contrary, in this work a decrease in *L*_50_ was observed. All of the above strengthens the argument that one of the key drivers behind the observed dynamics in hake maturation is environmental change. However, further research is required concerning the effect of fishing pressure, particularly the interaction between climate change and fishery exploitation, as the two are confounding factors at the geographic boundaries of the species’ spatial extent [84].

Describing the biological processes underlying hake population dynamics can assist in improving their assessment and management. It is worth noting that the data used in this study were limited to the eastern Ionian Sea and that these results should be interpreted with caution. Furthermore, as this analysis was based on female hake, investigating male *L*_50_ is in our future plans and may contribute to further exploring maturation trends in both sexes of the hake population in the study area. Our results highlight the importance of investigating changes over time and updating crucial population parameters such as size-at-maturity. This can be achieved by linking environmental changes with population dynamics at a regional scale.

## 5. Conclusions

This work documented a decline in the size-at-maturity of European hake in the eastern Ionian Sea throughout a period of 40 years coinciding with an important environmental regime shift, which took place in the late 1990s/early 2000s, with most of the studied environmental variables increasing from this period on. The examined environmental stressors expressing this shift (a combination of relevant biotic and abiotic factors: productivity, temperature, salinity, *pH*, etc.) explained 80% of the deviance in the hake size-at-maturity decrease during this period. As many fish stocks already experience fishery-induced evolution [82], further studies are necessary to explore whether this is a case of an environmental impact on a possibly already overexploited fish population.

## Figures and Tables

**Table 1:** List of environmental variables used in the present work.

Variable	Units	Description
*T*	°C	Sea surface temperature
*S*	psu	Salinity
*ssh*	m	Sea surface height
*grossPP*	tons C/day	Gross primary production
*Z4c*	mg C/m^3^	Mesozooplankton C
*Z5c*	mg C/m^3^	Microzooplankton C
*chl4*	mg/m^3^	Microphytoplankton Chl
*pH*	−log[H+]	Degree of acidity/alkalinity
*O*	mmol/m^3^	Dissolved oxygen
*ben_sus*	mg/m^2^	Suspension/filter feeders, macrobenthos
*ben_DOC*	mg/m^2^	Benthic dissolved organic carbon
*MLD*	m	Mixed layer depth

**Table 2:** Contribution of environmental (physicochemical and biological) variables to PC1.

Variable	% PC1 Contribution
*Z5c*	10.2
*pH*	10.1
*grossPP*	9.5
*chl4*	9.5
*ben_sus*	9.2
*ben_DOC*	9.2
*S*	9.1
*ssh*	8.4
*T*	8.2
*Z4c*	7.5

**Table 3:** Generalized additive model results for the environmental stressor (PC1) affecting European hake size-at-maturity (*L*_50_) in the eastern Ionian Sea during 1983–2021.

Family: Gamma	Link Function: log			
Formula: *L*_50_ ~ *s*(PC1, *k* = 7)				
Parameter	Edf	Ref.df	*F*	*p*-Value
*s*(PC1)	5.392	5.859	6.907	0.00313
*R*-sq.(adj) = 0.696				
Deviance explained = 80.2%				
GCV = 0.00085265				
Scale est. = 0.00053114, n = 17				

*s*: smooth function represented using penalized regression splines; df: degrees of freedom; Edf: estimated degrees of freedom; *F*: *F*-ratio test score; *p*-value: refers to the *p*-values from an ANOVA *F*-ratio test.

## Data Availability

The data presented in this study are available on request from the corresponding author. The data are not publicly available due to relevant limitations imposed from the authority (Greek Ministry of Agricultural Development and Food) financing the Greek Fisheries Data Collection Framework.

## Supplementary Materials

The following supporting information can be downloaded at: https://www.mdpi.com/article/10.3390/ani14010061/s1, Table S1: Research projects conducted by the IMBRIW (Institute of Marine Biological Resources and Inland Waters—HCMR, Greece), the data of which were used for the present work (OTB: bottom trawl; GNS: set gillnets; GTR: trammel nets; GTN: combined gillnets–trammel nets; LLS: stationary longlines). Table S2: Total number of analyzed female European hake individuals per year in the eastern Ionian Sea. Table S3: Full list of environmental variables investigated for the period 1960–2021. Figure S1: Distance correlation plot of the investigated environmental variables (yellow-green points should be considered to be not highly correlated). Table S4: Two-sample Kolmogorov–Smirnov test results of length frequency distribution between European hake samples, collected using bottom trawl (OTB) and using all gear combined, per year (*p*-value < 0.05: statistically significant difference). Figure S2: Time trend of PCA scores for the two principal components (high: 1983–2021; low: 1960–2021). Figure S3: Maturity ogives of female European hake in the eastern Ionian Sea, showing the probability of mature individuals. 95% confidence intervals are displayed as red lines. Table S5: Estimated size-at-maturity (*L*_50_) (mm) and its confidence intervals (C.I.) (mm) for female European hake for the studied years in the eastern Ionian. Figure S4: Traffic light plots for the 12 selected environmental variables from the eastern Ionian Sea during the period 1960–2021. Temporal development of values sorted according to their loadings on the first principal components (PC1). Figure S5: Principal component analysis (PCA) of the environmental variables. Unscaled time trajectory of PC1 vs. PC2. PCA conducted for the eastern Ionian Sea environmental variables covering the period 1960–2021. Figure S6: Evolution of Greek fleet in terms of capacity (in gross tonnage) in the period 1970–2021.
