# Supplementary material for: Decline in Size-at-Maturity of European Hake in Relation to Environmental Regimes: A Case in the Eastern Ionian Sea
Source: Animals (Basel). 2023 Dec 23;14(1):61. doi: 10.3390/ani14010061 (PMC10777906; doi:10.3390/ani14010061)
Supplement: Supplementary file 1 [file animals-14-00061-s001.zip › animals-2711555-supplementary.pdf]

Supplementary material of the paper "Decline in Size-at-Maturity of European Hake in Relation to Environmental Regimes: A Case in the Eastern Ionian Sea".

Table S1. Research projects conducted by the IMBRIW (Institute of Marine Biological Resources and Inland Waters - HCMR, Greece), the data of which were used for the present work. (OTB: Bottom Trawl; GNS: Set gillnets; GTR: Trammel nets; GTN: Combined gillnets-trammel nets; LLS: Stationary longlines)

Project	Sampling Year	Sampling Gear
Ionio-Patraikos	1983-1985	OTB
INTERREG II GREECE-ITALY	1999, 2000	OTB
RESHIO	2000, 2001	OTB
Artificial Reefs	2004	OTB
MEDITS	1995, 1999-2001, 2004-2006, 2008	OTB
NDCP (National Data Collection Programme)	2004-2006, 2008	OTB, GNS, GTR, LLS
COCONET	2014	OTB
DCF (Data Collection Framework)	2014, 2016, 2018-2021	OTB, GNS, GTN, GTR, LLS

Table S2. Total number of analyzed female European hake individuals per year in the eastern Ionian Sea.

Year	Total Number
1983	408
1984	505
1985	421
1995	159
1999	126
2000	494
2001	204
2004	697
2005	434
2006	576
2008	622
2014	476
2016	392
2018	429
2019	471
2020	449
2021	278
**Total**	**7141**

Table S3. Full list of environmental variables investigated for the period 1960-2021.

Variable name	Units	Description
T	^oC	Sea Surface Temperature
S	psu	Salinity
ssh	m	Sea Surface Height
grossPP	tonsC/d	Gross Primary Production
netPP1	$mgC/m^3/d$	Net Primary Production by diatoms
P1c	mgC/m^3	Diatoms C
P2c	mgC/m^3	Nanophytoplankton C
P3c	mgC/m^3	Picophytoplankton C
P4c	mgC/m^3	Microphytoplankton C
Z4c	mgC/m^3	Mesozooplankton C
Z5c	mgC/m^3	Microzooplankton C
Z6c	mgC/m^3	Heterotrophic Nanoflagellates C
B1c	mgC/m^3	Bacteria C
chl1	mg/m^3	Diatoms Chl
chl2	mg/m^3	Nanophytoplankton Chl
chl3	mg/m^3	Picophytoplankton Chl
chl4	mg/m^3	Microphytoplankton Chl
pH	-log[H+]	Degree of acidity/alkalinity
O	$mmol/m^3$	Dissolved Oxygen
ComProd	tonsC/d	Community production
MLD	m	Mixed layer depth
ben_DIC	$mmol/m^2$	Benthic Carbon Dioxide
ben_N	$mmol/m^2$	Benthic Nitrate - Aerobic Layer
ben_NH4	$mmol/m^2$	Benthic Ammonium - Aerobic Layer
ben_P	$mmol/m^2$	Benthic Phosphate - Aerobic Layer
ben_Si	$mmol/m^2$	Benthic Silicate - Aerobic Layer
ben_O	$mmol/m^2$	Benthic Oxygen
ben_dep	mg/m^2	Deposit Feeders, Macrobenthos C
ben_sus	mg/m^2	Suspension / Filter Feeders, Macrobenthos C
ben_DOC	mg/m^2	Benthic Dissolved Organic Carbon
optChl	mgC/m^2	Chlorophyll-a averaged over the optical depth
optDepth	m	Optical Depth (i.e. depth over which the light reduces by factor e)

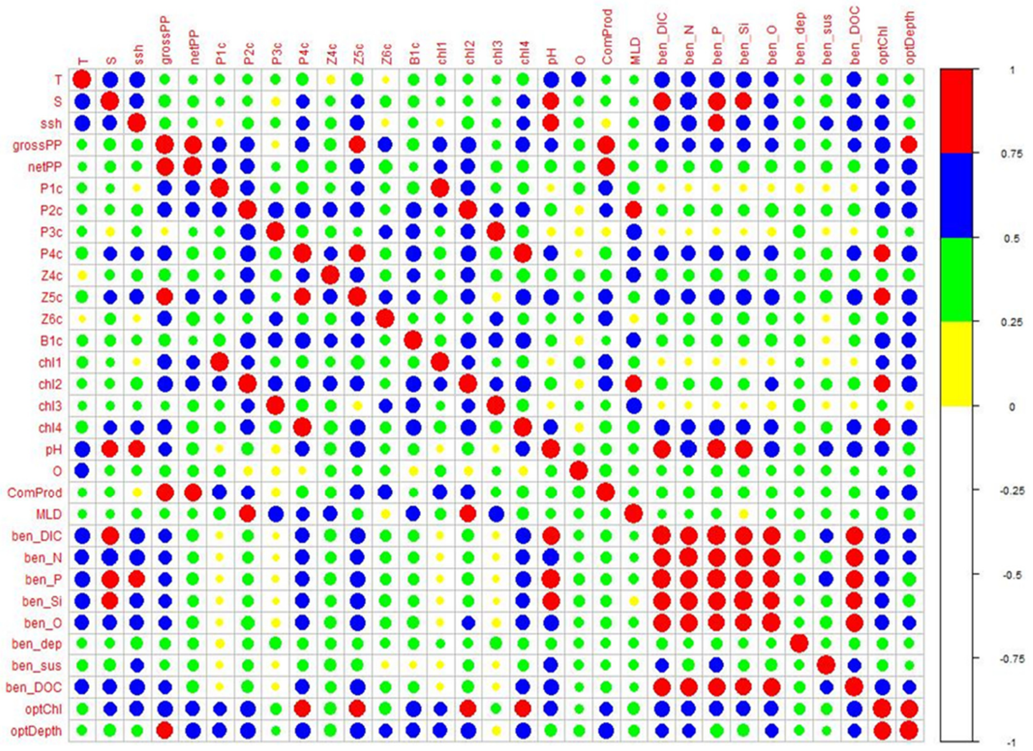

Figure S1. Distance correlation plot of the investigated environmental variables (yellow-green points should be considered as not highly correlated).

Table S4. Two-Sample Kolmogorov-Smirnov test results of length frequency distribution between European hake samples, collected by bottom trawl (OTB) and by all gears combined, per year (*P-value* < 0.05: statistical significant difference).

**Year**	**OTB – All gears** ***P-value***
2004	0.88
2005	0.99
2006	0.06
2008	0.06
2014	0.51
2016	0.76
2018	0.74
2019	0.48
2020	0.95
2021	0.17

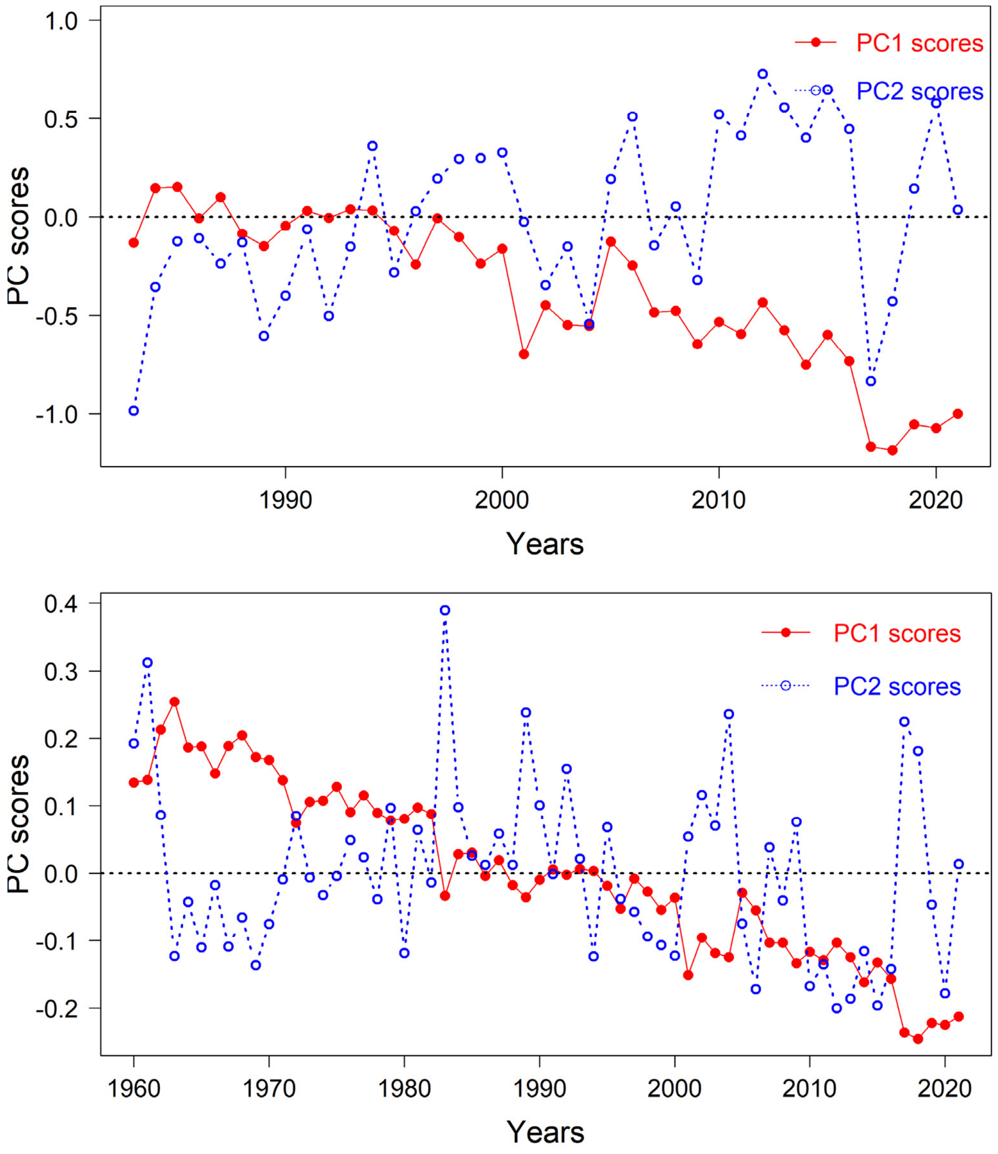

Figure S2. Time trend of PCA scores for the two principal components (Up: 1983-2021; low: 1960-2021).

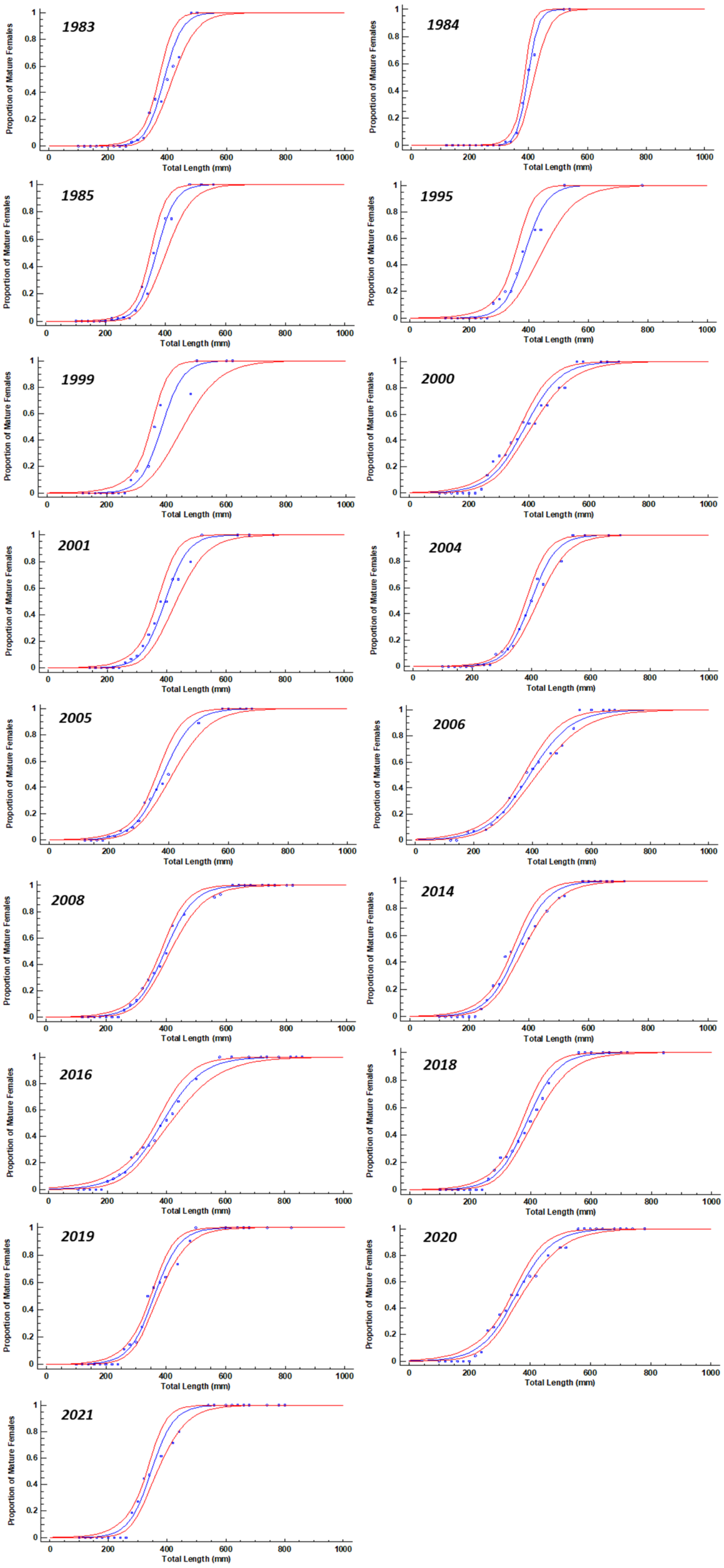

Figure S3. Maturity ogives of female European hake in the eastern Ionian Sea, showing the probability of mature individuals. 95% Confidence Intervals are displayed in red lines.

Table S5. Estimated size-at-maturity (L_{50}) (mm) and its confidence intervals (C.I.) (mm) of female European hake for the studied years in the eastern Ionian.

Year	L_{50} (mm)	Lower 95% C.I. (mm)	Upper 95% C.I. (mm)
**1983**	389.6	371.7	417.4
**1984**	397.3	385.3	418.3
**1985**	369.8	350.0	401.7
**1995**	387.6	356.4	444.5
**1999**	383.9	348.8	455.0
**2000**	386.1	368.9	407.5
**2001**	394.8	369.3	432.4
**2004**	399.1	381.4	423.1
**2005**	382.4	361.3	413.5
**2006**	388.8	369.8	413.5
**2008**	396.0	382.4	413.6
**2014**	362.5	346.7	382.2
**2016**	384.1	364.7	410.9
**2018**	389.0	372.3	411.1
**2019**	361.9	350.2	376.0
**2020**	355.3	340.4	373.2
**2021**	345.9	331.1	366.8

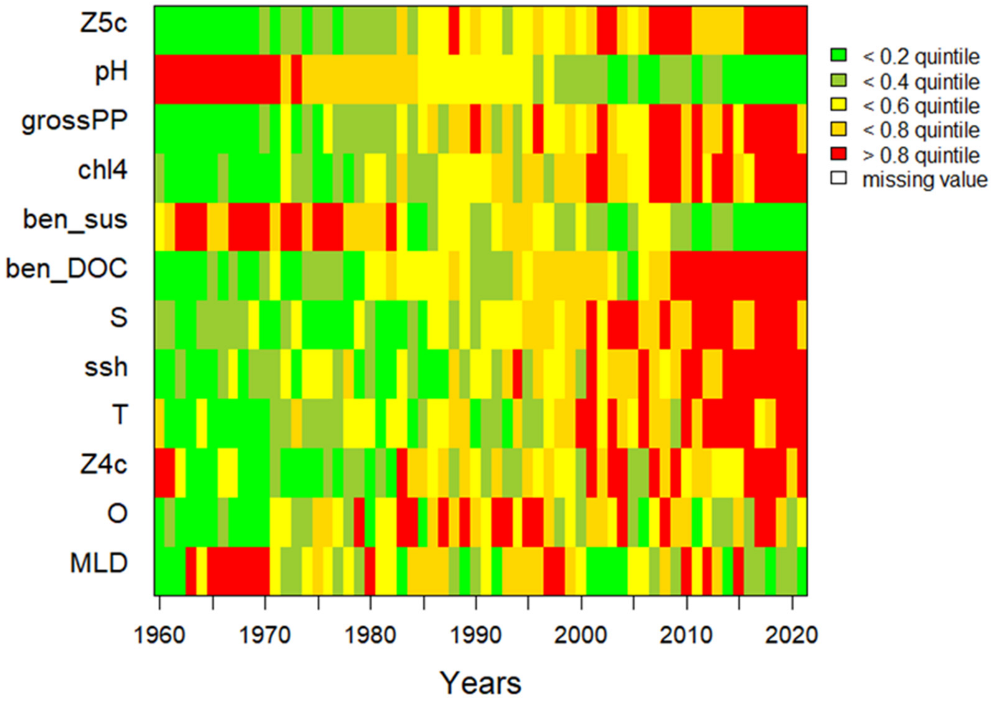

Figure S4. Traffic light plots for the 12 selected environmental variables from the eastern Ionian Sea during the period 1960-2021. Temporal development of values sorted according to their loadings on the first principal components (PC1).

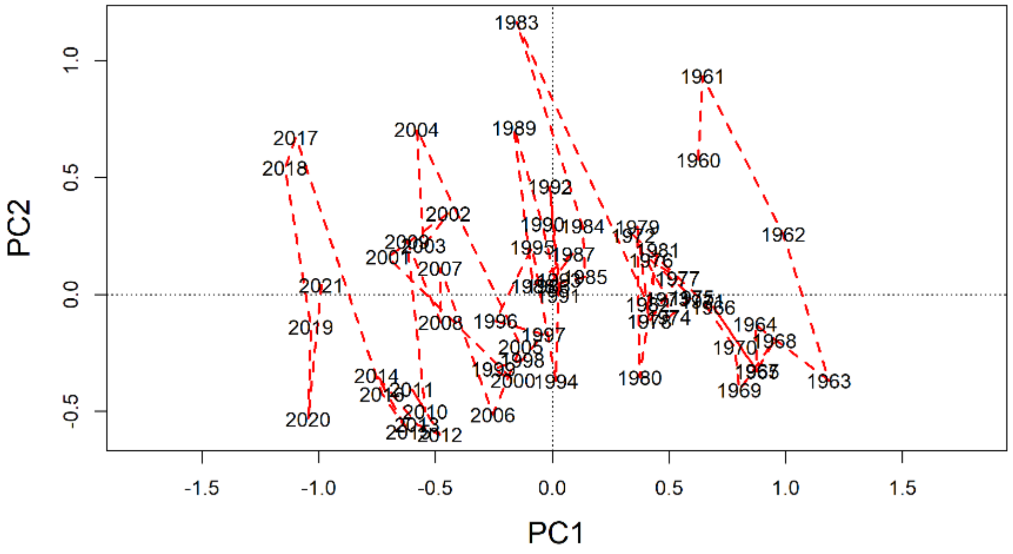

Figure S5. Principal Component Analysis (PCA) of the environmental variables. Unscaled time trajectory of PC1 vs PC2. PCA conducted for the eastern Ionian Sea environmental variables covering the period 1960-2021.

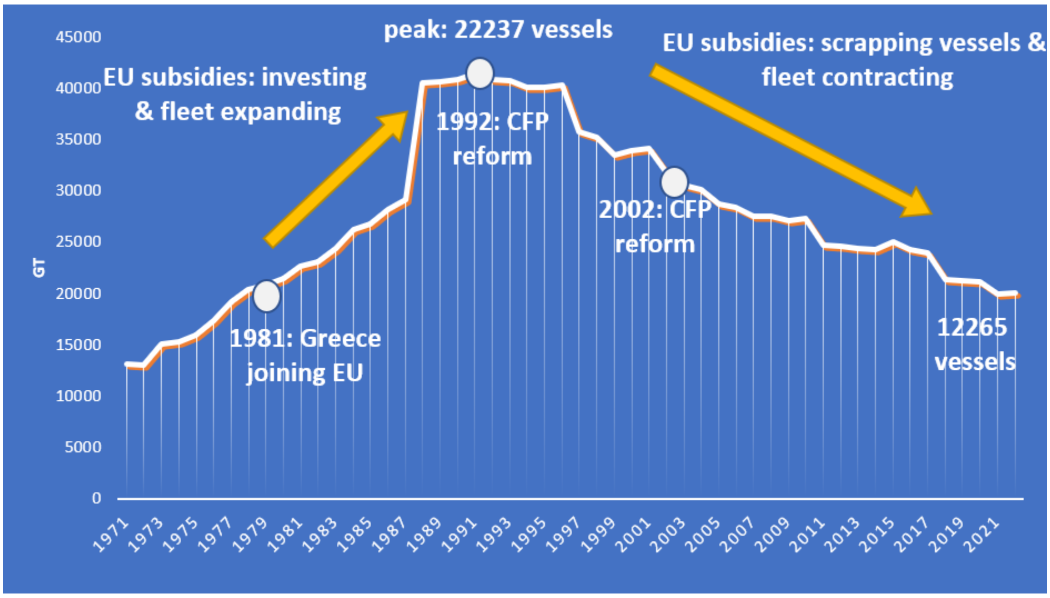

Figure S6. Evolution of Greek fleet in terms of capacity (in Gross Tonnage) the period 1970-2021.
